# Establishment and characterization of novel spontaneously immortalized larval cell lines from sablefish *Anoplopoma fimbria*

**DOI:** 10.1007/s11626-024-00975-3

**Published:** 2024-09-30

**Authors:** Eric R. Friesen, Amy K. Long, Kyle A. Garver

**Affiliations:** Pacific Biological Station, Fisheries and Oceans Canada, Pacific Biological Station 3190 Hammond Bay Rd., Nanaimo, BC V9T6N7 Canada

**Keywords:** Aquaculture, Cell line, Development, Larva, Sablefish, Pacific Ocean

## Abstract

Sablefish *Anoplopoma* *fimbria* is a groundfish of the North Pacific Ocean typically found in sea floor habitat at depths to 2700 m. Prized as a food fish with exceptionally high market value, sablefish aquaculture has been sought to provide a sustainable source of this fish to meet market demands. While commercial culture has successfully produced market-sized fish in Pacific coastal environments, production has been hampered by disease and the overall lack of information on sablefish health and immunology. To begin to address these knowledge gaps, herein we describe the isolation and characterization of spontaneously immortalized sablefish larval cell lines (AFL). Six sublines were established from pools of early yolk-sac larvae, while attempts to develop tissue-specific–derived cell lines were unsuccessful. The six yolk-sac larval cell lines each display two morphologies in culture, an elongated fibroblast-like cell type, and a rounded squamous or epithelial-like cell type. Cytogenetic characterization suggests that both cell types are diploid (2n = 48) with 24 pairs of chromosomes, 23 pairs of autosomes, and 1 pair of sex chromosomes. A small proportion (11%) of AFL cells display tetraploidy. Incubation temperature and medium composition experiments revealed HEPES buffered L-15 media containing 10–20% FBS at temperatures between 15 and 18° C yielded optimal cell growth. These growth characteristics suggest that sablefish larval cells display a robustness for varying growth conditions. The establishment of AFL cell lines provides a foundational tool to study the physiology, health, immunology, and cell and molecular biology of sablefish.

## Introduction

Sablefish *Anoplopoma fimbria* (Pallas 1814) also known as black cod, or butterfish, is a groundfish of the North Pacific Ocean with a broad distribution from California through the Bering Sea and Aleutian Islands into coastal Japan (Sasaki 1985; Kendall and Matarese 1987). *Anoplopoma fimbria* spawn in coastal waters near the surface and deposit eggs and larva near inshore waters of depths to 300 m (Sasaki 1985; Kendall and Matarese 1987; DFO 2018). As *A. fimbria* develop, the larva and juveniles are found in inshore waters until they migrate to deeper waters, with adult fish inhabiting the ocean floor at depths to 2700 m (Sasaki 1985; Kendall and Matarese 1987; DFO 2018). Larval *A. fimbria* range in size from 1 to ~ 30 mm and juveniles from ~ 30 to ~ 70 cm, and adult fish may reach in excess of 1-m fork length (Sasaki 1985; Kendall and Matarese 1987; Fujiwara and Hankin 1988; Shaw 1993; Guzman *et al*., 2017; Goetz *et al*. 2021).

*Anoplopoma fimbria* in captivity rapidly increase biomass and are capable of growing at rates of > 2 mm d^−1^ and > 3 g d^−1^ (Kendall and Matarese 1987; Cook *et al*. 2018; Krieger *et al*. 2019; Krieger *et al*. 2020; Goetz *et al*. 2021), making them well suited for aquaculture. In addition, due to their rich oily flesh, *A. fimbria* are highly demanded as a food fish with a high market value. *Anoplopoma fimbria* aquaculture has been successful at producing market size fish; however, to date, the industry remains small with limited capacity as culture efforts are often hampered by disease and inadequate prevention and treatment interventions (Goetz *et al*. 2021).

*Anoplopoma fimbria* are known to be susceptible to bacterial pathogens including members of the *Vibrionaceae* family, *Renibacterium salmoninarum*, and *Aeromonas salmonicida* (Bell *et al*. 1990; Arkoosh and Dietrich 2015; Arkoosh *et al*. 2018; Goetz *et al*. 2021). It has also been shown that *A. fimbria* are resistant to infection by the aquatic viruses infectious hematopoietic necrosis virus (IHN) and viral hemorrhagic septicemia virus (VHS) (Traxler 2004). The susceptibility of *A. fimbria* to other aquatic viruses and bacteria remains an important area of further research. While a recent publication of the chromosome-level genome of *A. fimbria* (Flores *et al*. 2023) will undoubtedly facilitate our understanding of *A. fimbria*, in vitro models for this species are limited.

To date, 945 fish cell lines have been documented in Cellosaurus (Release 49, May 2024) (Bairoch A., 2018), representing only a small fraction of known fish species (Bols *et al*. 2023). Currently, there are no cell lines from *A. fimbria* and only a few cell lines from fish species of the Northern Pacific Ocean (Ganassin *et al*. 1999; Pham *et al*. 2020). In the study herein, we used sablefish yolk-sac larvae to develop several *A. fimbria* cell lines (denoted AFL) with different cell morphologies and possibly the capacity to produce melanin. Besides being a first for this species, these cell lines join PHL from the Pacific herring (Ganassin *et al*. 1999) as being the only ones from completely marine fish of the Northern Pacific Ocean, and provide a foundational tool to study the physiology, health, immunology, and cell and molecular biology of sablefish.

## Materials and methods

### Fish source and health screening

As a source of tissue for cell line development, *Anoplopoma fimbria* of various lifestages, ages, and rearing conditions (as summarized in Table 1) were obtained from Golden Eagle Sablefish hatchery, British Columbia, Canada. The fish obtained were offspring of captive wild broodstock. All fish used in this study were sacrificed in 100 mg/L tricaine methanesulphonate (MS 222; Syndel, Nanaimo, Canada) and subsequently examined for external signs of disease. Fish from each lifestage were screened for virus using standard tissue culture methodologies (Long *et al*. 2021). Briefly, for both yolk-sac and late larvae, 20 individuals of each were pooled and homogenized, while kidney and spleen were harvested aseptically from the 119 (*n* = 5) and 199 (*n* = 2) d post-fertilized juveniles. All whole larvae or kidney/spleen tissue pools were inoculated in duplicate onto monolayers of chinook salmon embryo (CHSE-214; ATCC CRL-1681), epithelioma papulosum cyprini (EPC; ATCC CRL-2872), bluegill fry 2 (BF2; ATCC CCL-91), and striped snakehead (SSN-1; ECACC 96,082,808) cells in 24-well cell culture plates. Inoculated plates were incubated at 15°C for 3 wk and monitored two to three times weekly for cytopathic effects (CPE).

**Table 1.** Age and lifestage of *Anoplopoma fimbria* from which primary cells were produced

Lifestage	Age (d post-fertilization)	Size (g)	Seawater rearing conditions	
			Salinity (ppt)	Temperature (°C)
Yolk-sac larva	45	0.004	30	5.0
Late larva	57	0.011	26	10.0
Juvenile	119	1.0	26	14.3
Juvenile	199	70.0	26	15.3

### Primary cell culture, subculture, and establishment of AFL cell lines

To initiate primary cultures, tissues were harvested aseptically from sablefish immediately after euthanasia. Pectoral fin, gill, liver, spleen, brain, and heart were collected from ~ 70 g of juvenile fish. Whole larval fish were pooled into groups of 15–20 fish and processed. Tissue was placed in sterile petri dishes, resuspended in 500 μL phosphate buffered saline (PBS; ThermoFisher, Waltham, MA) with 2% (v/v) antibiotic/antimycotic (Gibco, Grand Island, NY), and minced to a fine paste using sterile scalpel blades. The resulting tissue masses were washed three times in antibiotic/antimycotic-PBS and digested in a 0.25% Trypsin (Gibco﻿) + antibiotic/antimycotic-PBS solution for 20 min, vortexing every 10 min. This 0.25% trypsin digest was repeated three times with cell suspension supernatant harvested and retained between each digest. The resultant three cell suspensions were pooled and centrifuged at 200 × *g* for 10 min at 4°C. The cell pellet was resuspended in 500 μL of media composed of L15 media (Gibco﻿) supplemented with 15% fetal bovine serum (FBS) (Gibco﻿), 2 × antibiotic/antimycotic, and 14 mM HEPES buffer (Gibco﻿) (denoted hereafter as AFL media). The resultant cell suspension was enumerated using a Countess automated cell counter (Invitrogen, Carlsbad, CA) and subsequently brought to a final volume of 1 mL. The entire 1-mL cell suspension was seeded into an individual well of a 12-well plate resulting in approximately 1 × 10^6^ to 2.5 × 10^6^ cells per well. Cells were incubated at 18°C without additional CO_2_ and monitored daily for 25 d for monolayer formation or cell death. In the event of cell monolayer formation, subcultivation was achieved by adding 0.05% trypsin solution to each well and incubated for 5 min, after which trypsin was inactivated by addition of AFL media. Cell suspension was then diluted 1:2 (v/v) in AFL media into two wells of a 12-well cell culture plate and monitored for monolayer formation or cell death. Cells which remained viable and formed a monolayer were again trypsin digested and passaged 1:1 (v/v) in AFL media into 25-cm^2^ cell culture flasks. Upon monolayer formation in 25-cm^2^ flasks, cells were passaged and split 1:2 (v/v) in AFL media, seeded into 75-cm^2^ cell culture flasks, and monitored continuously for monolayer formation or cell death.

### Polyclonal AFL cell line growth optimization/Optimal growth temperature

Four 75-cm^2^ cell culture flasks of polyclonal AFL line 6A at passage 20 were harvested by standard 0.05% trypsin digest, pooled, and seeded into 12-well plates at 9.36 × 10^5^ ± 5.68 × 10^4^ cells mL^−1^ per well. Cells were incubated at 10 ± 0.2°C, 15 ± 0.74°C, 18 ± 0.24°C, and 20 ± 0.56°C for a total of 216 h. At 0, 72, 144, and 216 h, five wells of each temperature treatment (i.e., two 12-well plates required per temperature) were washed twice with antibiotic/antimycotic-PBS, trypsinized, and centrifuged at 400 × *g* at 4°C for 5 min. The supernatant was discarded and the cell pellet was resuspended in 500 μL of AFL media. The cell suspension was enumerated using a Countess automated cell counter (Invitrogen) with standard 0.4% trypan blue live/dead staining (Invitrogen, Eugene, OR).

### Optimal medium serum composition

To determine the optimal medium composition, four 75-cm^2^ cell culture flasks of polyclonal AFL line 6A were harvested at passage 14. Cell suspension was pooled and seeded into 12-well plates at 2.72 × 10^5^ ± 5.26 × 10^4^ cells mL^−1^ per well. Cells were overlaid with AFL media that differed in FBS (Gibco﻿) at a final concentration of 0%, 5%, 10%, 15%, or 20% and incubated at 18 °C for a total of 216 h. At 0, 72, 144, and 216 h, cells were harvested from each treatment. Samples were harvested and enumerated as described in the section “Optimal growth temperature”.

### Single-cell isolation by limiting dilution

To establish a monoclonal culture from mixed cell populations, limiting dilution was conducted following methods as described elsewhere (Marjan *et al*. 2021). Briefly, AFL cells of polyclonal lines 5, 10, and 12 at passages 18, 6, and 17 respectively were harvested and cells centrifuged at 400 × *g* at 4°C for 5 min and resuspended in 1 mL of AFL media. Each AFL line was enumerated by automated cell counter and diluted to 1 × 10^6^ cells mL^−1^ in AFL media. The resulting cell suspensions were further diluted to yield ~ 1 cell per 100 μL of AFL media. A 100-μL aliquot of each cell suspension was then seeded into flat bottom 48-well cell culture plates (Nunc, Roskilde, Denmark), incubated at 18 °C, and monitored twice weekly for monolayer formation. If > 50% coverage was obtained, the cell monolayer was trypsinized, harvested, and reseeded 1:1 (v/v) into a six-well cell culture plate and monitored twice weekly for growth. Once monolayer coverage reached > 75% of the well, cells were trypsinized, harvested, and reseeded 1:1 (v/v) into a 25-cm^2^ cell culture flask and monitored twice weekly for monolayer formation. Thereafter, monolayers were passaged as normal and maintained in culture indefinitely.

### Chromosome analysis

To perform chromosome analysis, AFL line 6A at passage 7 was grown to 80% confluency and the growth medium was replaced with fresh AFL media. Cells were treated with 10 μg mL^−1^ colchicine (Sigma-Aldrich, Ward Hill, MA) and incubated overnight. Treated cells were then harvested and centrifuged at 200 × *g* at 4°C for 10 min. The supernatant was discarded and the cell pellet was resuspended in 0.075 M KCl and incubated for 30 min at 18°C and cells gently vortexed every 10 min. The cell suspension was centrifuged at 200 × *g* at 4°C for 5 min and the cell pellet was resuspended in 5 mL of fresh Carnoy’s fixative (3 parts methanol:1 part glacial acetic acid) and incubated for 5 min at room temperature. Fixed cells were centrifuged at 200 × *g* at 4°C for 5 min and the supernatant was discarded and cells were resuspended in a fresh fixative. This wash was repeated three times with the final cell pellet resuspended in 5 mL of fixative. Fixed cells were then stored at 4°C. Upon use, cells were centrifuged as above and resuspended in 2 mL of fresh Carnoy’s fixative. To prepare the slides, 8 μL of cell suspension was dropped from a height of ~ 3 cm onto a clean microscope slide at room temperature. Slides were placed onto a floating metal tray, within a covered 50°C water bath for 70 s, after which the slides were air-dried for 5 min (Deng *et al*. 2003; Howe *et al*. 2014). Slides were then flooded with modified Giemsa stain (Sigma-Aldrich, St. Louis, MO) for 2 min, rinsed, dried overnight, and mounted with coverslips. Chromosome spreads (*n* = 100) were visualized and counted with a light microscope (Zeiss, White Plains, NY) at × 100 with oil immersion.

### Cryopreservation and revival

To determine optimal cryopreservation conditions, one 75-cm^2 ^cell culture flask each of polyclonal AFL cell lines 5A, 10A, and 11A was trypsinized, harvested, and centrifuged as described above. Working on ice, cell pellets were resuspended in 500 μL pre-chilled AFL media and augmented with 500 μL of one of the following cryoprotectants added dropwise to yield a final concentration of the following: 25% FBS, 50% FBS, 25% CTS Synth-a-Freeze (Gibco﻿), 50% Synth-a-Freeze, 5% DMSO (Sigma-Aldrich, Solon, OH), 70% glycerol (Sigma-Aldrich, St. Louis, MO), and 70% glycerol + 5% DMSO. Additionally, concentrations of 100% FBS and 100% Synth-a-Freeze were each evaluated by resuspending the cell pellet directly into either 1 mL of FBS or Synth-a-Freeze, respectively. Cell/cryoprotectant suspensions were aliquoted into 1.8 mL of external thread screw-cap cryovials (Nunc, Roskilde, Denmark) and placed in a Mr. Frosty™ (ThermoFisher) 1°C min^−1^ rate limited freezing container and chilled overnight at − 80°C. Vials were then sealed in Cryo-Flex Tube Wrapping (Nunc) and stored immersed in liquid nitrogen for 14. Cryopreserved cells were revived by removing vials from liquid nitrogen and immediately immersed in a 37°C water bath until the frozen mass dislodged from the walls of the cryovial. The dislodged cell mass was then decanted into a 25-cm^2^ cell culture flask containing AFL media prewarmed to 18°C and then incubated at 18°C as described above. Twenty-four hours post-thaw, cells were viewed on an inverted light microscope (Zeiss). For the viable cell cultures, the freezing medium was removed and replaced with fresh AFL media and subsequently monitored and maintained for up to 8 wk.

### Molecular confirmation of cell line origin

To confirm the species origin of AFL cells, a 750-bp fragment of the cytochrome oxidase subunit-1 (*co1*) gene was amplified by conventional PCR. Total DNA was isolated from each of the six polyclonal AFL lines (AFL-5A, AFL-6A, AFL-9A, AFL-10A, AFL-11A, and AFL-12A) at passage 18 using DNeasy Blood & Tissue kit (Qiagen, Hilden, Germany) following the manufacturer’s protocols. Partial fragments of the *co1* gene were amplified using primers designed for the *co1* sequence in fish (Ward* et al*. 2005; Ivanova *et al*. 2007). PCR amplification was performed with 12.4 μL nuclease-free water (Invitrogen, Grand Island, NY), 4 μL of 5 × Phusion high-fidelity polymerase buffer (New England Biolabs, Ipswich, MA), 0.4 μL of 10 mM dNTP’s (Invitrogen, Waltham, MA), 1 μL each of 10 μM forward and reverse primers (Ward *et al*. 2005; Ivanova *et al*. 2007), and 0.2 μL of 0.1 U/μL Phusion high-fidelity polymerase (New England Biolabs). Thermal cycling was as follows: 98°C for 2 min followed by 35 cycles of 98°C for 15 s, 59.5°C for 30 s, 72°C for 45 s, followed by a final 72°C extension for 10 min and a 4 °C indefinite hold. PCR products were separated by electrophoresis through 0.8% agarose gel stained with SYBR Safe (Invitrogen, Waltham, MA) at 90v for 1 h and visualized by UV light at 302 nm. PCR products were purified using ExoSAP-IT PCR product clean-up kit (Applied Biosystems, Waltham, MA). Purified amplicons were sequenced using BigDye 3.1 Sanger sequencing kit (Applied Biosystems, Waltham, MA) and sequences were read on a SeqStudio Genetic Analyzer (Applied Biosystems). Sequences were queried against databases in NCBI’s GenBank using BLASTn (NCBI, Bethesda, MD).

### Detection of Mycoplasma contamination and Gram stain

Each of the six polyclonal AFL lines (AFL-5A, AFL-6A, AFL-9A, AFL-10A, AFL-11A, and AFL-12A) at passage 22 was examined for *Mycoplasma* contamination using Mycosensor QPCR Assay Kit (Agilent, Cedar Creek, TX). Briefly, 1 mL of cell suspension was heated at 95°C for 5 min, followed by centrifugation at 14,000 × *g* for 1 min. Supernatant was collected and 100 μL was extracted and screened via qPCR according to the manufacturer’s instructions. Each of the six polyclonal AFL lines at passage 22 was examined for bacterial contamination by standard Gram stain protocol (Gerhardt *et al*. 1981). Briefly, 1 mL of cell culture supernatant from each AFL line was harvested aseptically. A 10-μL loopful of supernatant was heat fixed to clean a microscope slide and Gram stained. Stains were observed with a light microscope (Zeiss) at × 100 with oil immersion.

### Sex determination of polyclonal AFL lines

To determine the sex of each of the six polyclonal AFL cultures, a partial sequence of the gonadal soma-derived factor gene (*gsdf*) and upstream chromosomal DNA was amplified by conventional PCR. Total DNA was extracted from the following AFL lines using DNeasy Blood & Tissue kit (Qiagen, Hilden, Germany) following the manufacturer’s instructions: AFL-5A at passage 44, AFL-6A at passage 40, AFL-9A at passage 39, AFL-10A at passage 36, AFL-11A at passage 33, and AFL-12A at passage 44. Sex-specific partial *gsdf* sequences were amplified using two primer sets designed for the amplification of an X-specific or Y-specific region near *gsdf* (Rondeau *et al*. 2013). PCR amplification was performed with 12.4 μL nuclease-free water (Invitrogen, Grand Island, NY), 4 μL of 5 × Phusion high-fidelity polymerase buffer (New England Biolabs), 0.4 μL of 10 mM dNTP’s (Invitrogen, Waltham, MA), 1 μL each of 10 μM forward and reverse primers (Rondeau *et al*. 2013), and 0.2 μL of 0.1 U/μL Phusion high-fidelity polymerase (New England Biolabs). Thermal cycling was as follows: 98°C for 2 min followed by 40 cycles of 98°C for 15 s, 58.7°C for 30 s, 72°C for 60 s, followed by a final 72 °C extension for 10 min and a 4°C infinite hold. PCR products were separated by electrophoresis through 0.8% agarose gel stained with SYBR Safe (Invitrogen, Waltham, MA) at 100 v for 1 h and visualized by UV light at 302 nm.

### Histopathology

To better understand the health and developmental timing of *Anoplopoma fimbria* larval stages of larvae used to generate the AFL cell line, histopathology was performed on yolk-sac bearing *A. fimbria* larvae aged 27 and 48 dpf. Eight to 12 larvae were sacrificed in TMS/MS-222 (Syndel, Nanaimo, Canada), placed in histology cassettes, and transferred to 10% neutral buffered formalin. Post-fixation, larvae were dehydrated, clarified, and blocked in paraffin for routine histological preparation. Larvae were sectioned at 4 μm, stained with hematoxylin and eosin, dehydrated with xylene, and mounted with a glass coverslip. Histopathology was performed by a single pathologist (GDM) who was not provided any information about the larvae prior to observation. Digital photomicrographs were processed as follows: white balance; uniform background; and live stitching of multiple fields into whole images was done using a MIchrome 6 digital camera coordinated with Mosaic 2.1 software.

### Statistical analysis

All statistical analyses were performed using GraphPad PRISM version 10.2.0 (GraphPad Software, Boston, MA). Differences in cell count between treatment groups within a timepoint were analyzed by one-way ANOVA followed by Tukey’s multiple comparisons post hoc test. Data are represented as the mean ± 1 standard deviation. Statistical differences were considered significant if *P* < 0.05.

### Ethical statement

The care and use of all animals used in this study complied with Canadian Council on Animal Care animal welfare laws, guidelines, and policies.

## Results

### Fish health screening

All fish were free of external signs of disease. All fish assayed by tissue culture were negative for CPE on all cell lines.

### Primary cell culture, subculture, and establishment of AFL cell line

**Figure 1.**
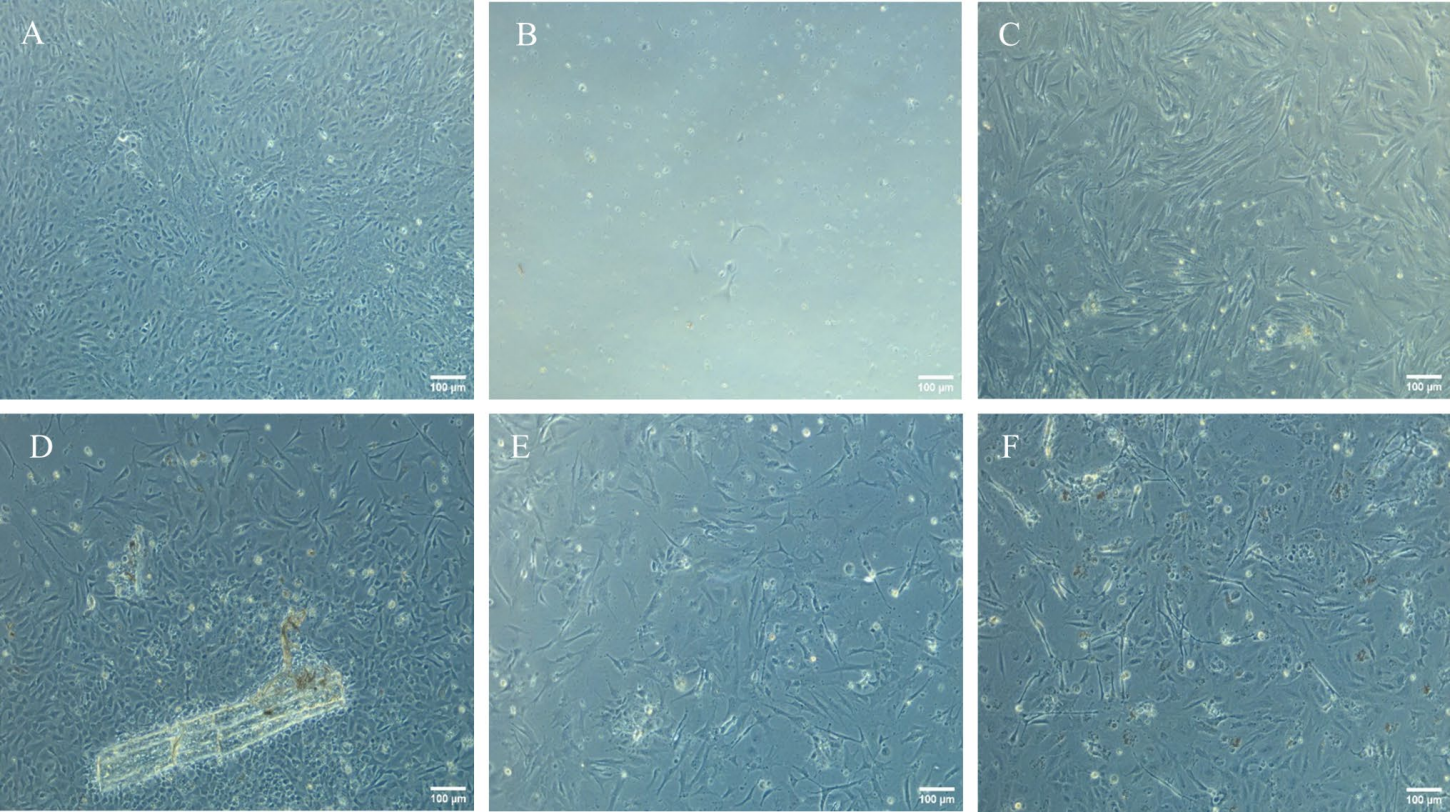
Primary sablefish, *Anoplopoma fimbria* cell populations remaining from either trypsin digested juvenile (199-d post-fertilization) tissues (*A–D*) or whole larvae (*E*, *F*): (*A*) brain tissue after 9 d in culture, (*B*) liver tissue after 9 d in culture, (*C*) heart tissue after 15 d in culture, (*D*) pectoral fin tissue after 9 d in culture (note the large piece of undigested fin cartilage), (*E*) whole late larva after 9 d in culture, and (*F*) yolk-sac larva after 9 d in culture. Phase contrast microscopy at × 100 magnification.

**Figure 2.**
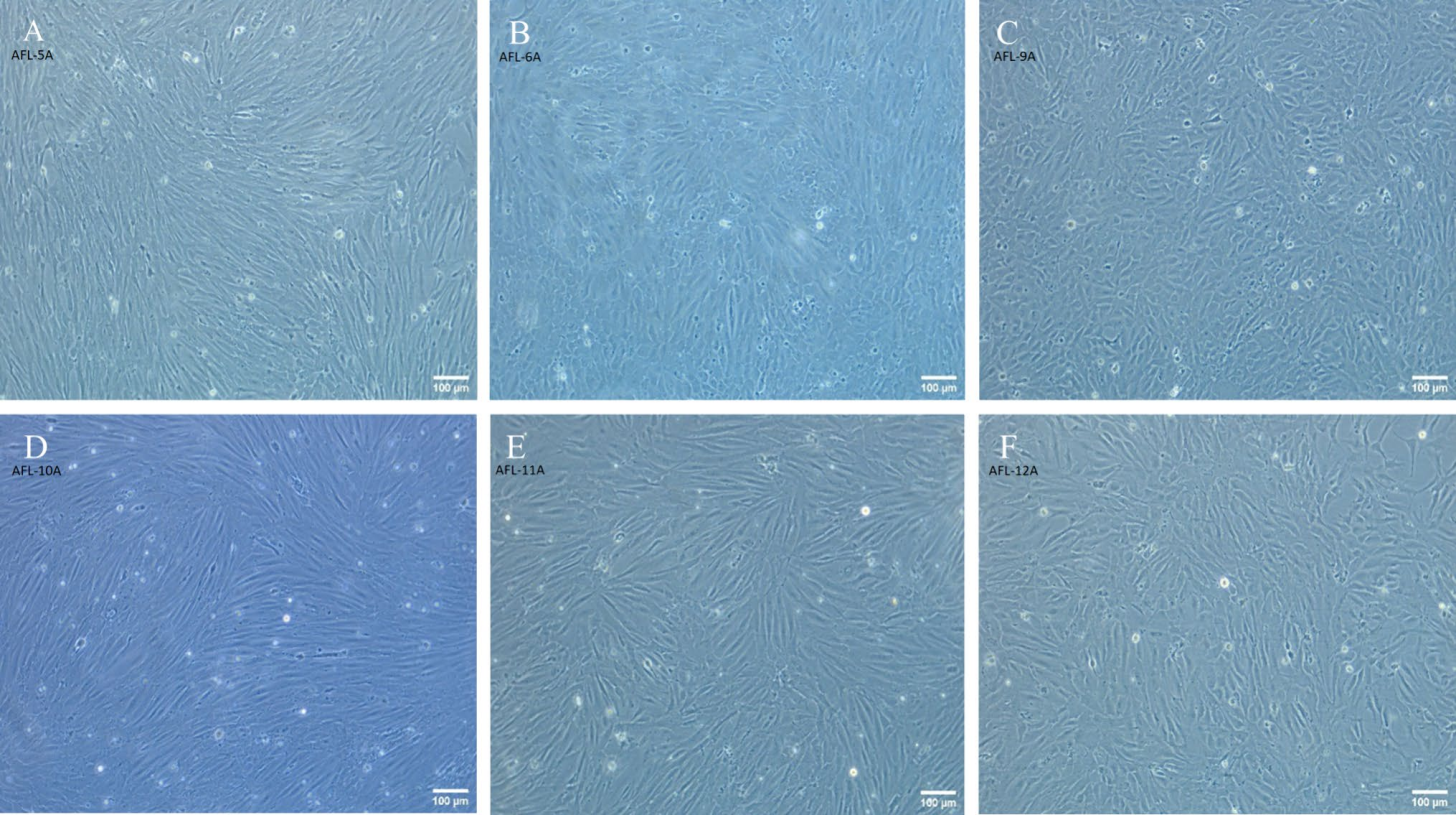
Morphologies of AFL cell lines. (*A)* AFL cell line 5A at passage 20 displaying mixed proportions of epithelial-like and fibroblast-like morphologies, (*B*) AFL cell line 6A at passage 19 displaying a predominantly epithelial-like morphology, (*C*) AFL cell line 9A at passage 23 displaying a predominantly epithelial-like morphology, (*D*) AFL cell line 10A at passage 20 displaying a predominantly fibroblast-like morphology, (*E*) AFL cell line 11A at passage 20 displaying a mixed proportion of epithelial-like and fibroblast-like morphologies, (*F*) AFL cell line 12A at passage 19 displaying predominantly epithelial-like morphology. Phase contrast microscopy of AFL cells at × 100 magnification.

**Figure 3.**
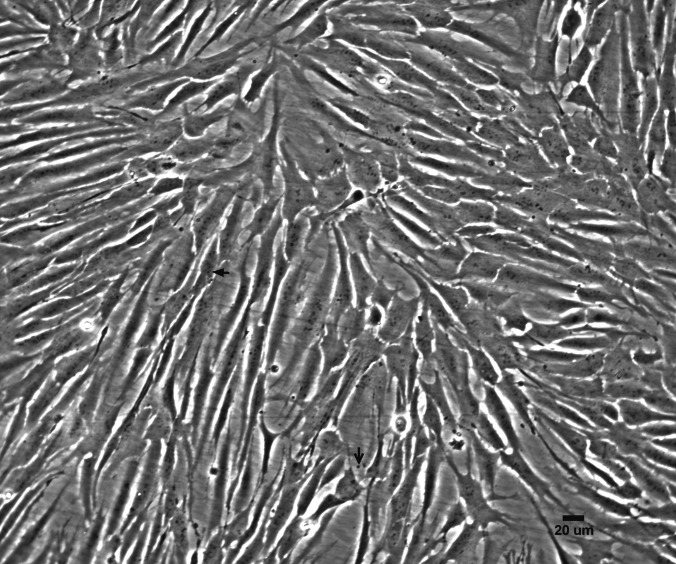
Presence of small black granules in cells (*filled arrow*) and the supernatant (*open arrow*) of AFL-6A grown in a 25-cm^2^ culture flask.

At 24-h post-plating, cells from all homogenized progenitor tissue inoculums displayed adherence to culture flasks, with some forming monolayers as early as 9 d (Fig. 1). Nonetheless, cultures of cells isolated from gills were the first to perish followed closely by liver, spleen, and brain. Similarly, all cultures isolated from late larvae perished concurrently though due to not cell death but rather bacterial colonization. The in vitro survivability of cells from each of the various tissue homogenate inoculums is summarized in Table 2. Among the tissue inoculums, only the yolk-sac larval lifestage yielded an established, continuously growing culture while all other tissue types resulted only in the establishment of primary cultures which survived for 9 to 52 d. Among the yolk-sac larval inoculations, 60% (18/30) were deemed unsuitable for passage within 24 d of post-initial seeding, while the remaining 12 inoculums were capable of being subcultured. By day 37, six of the 12 larval cell populations perished, resulting in six successful lines denoted as AFL-5A, AFL-6A, AFL-9A, AFL-10A, AFL-11A, and AFL-12A (Fig. 2*A*–*F*). Post 37 d, each of the six lines was maintained in 25-cm^2^ flasks and passed 1:10 when monolayers reached > 80% confluency. By day 79 (4^th^ passage), AFL lines exhibited sufficient growth and density to pass (1:5) into 75-cm^2^ culture flasks with subcultivation occurring every 7–10 d thereafter. Each of the six cell lines consisted of mixed populations of cells defined by two main morphologies, a rounded epithelial-like cell type (Fig. 2*B*, *C*, *F*) and an elongated fibroblast-like cell type (Fig. 2*A*, *D*, *E*). While some of the lines had a higher preponderance towards one of the cell morphologies, none of the lines contained solely one cell morphology and remained mixed populations for ~ 40 subcultivations. Across the six lines, AFL-6A demonstrated the most stable post-passage recovery particularly during the first five passages and was therefore selected as the candidate AFL line for further growth optimization and characterization. All AFL cell lines contained small black granules visible in cells and in the culture supernatant of AFL cells grown in culture flasks (Fig. 3). These granules, typically 2–3 μm in size, were initially thought to be contamination by bacteria or *Mycoplasma*; however, Gram stain and mycoplasma qPCR results were both negative.

**Table 2.** Viability duration for *Anoplopoma fimbria* primary cell cultures in vitro

Tissue origin of primary culture	Survival time (d)
Gill	9
Spleen	15
Liver	15
Brain	20
Heart	28
Fin	52
Late larva	15
Yolk-sac larva	> 365

### Single-cell isolation by limiting dilution

**Figure 4.**
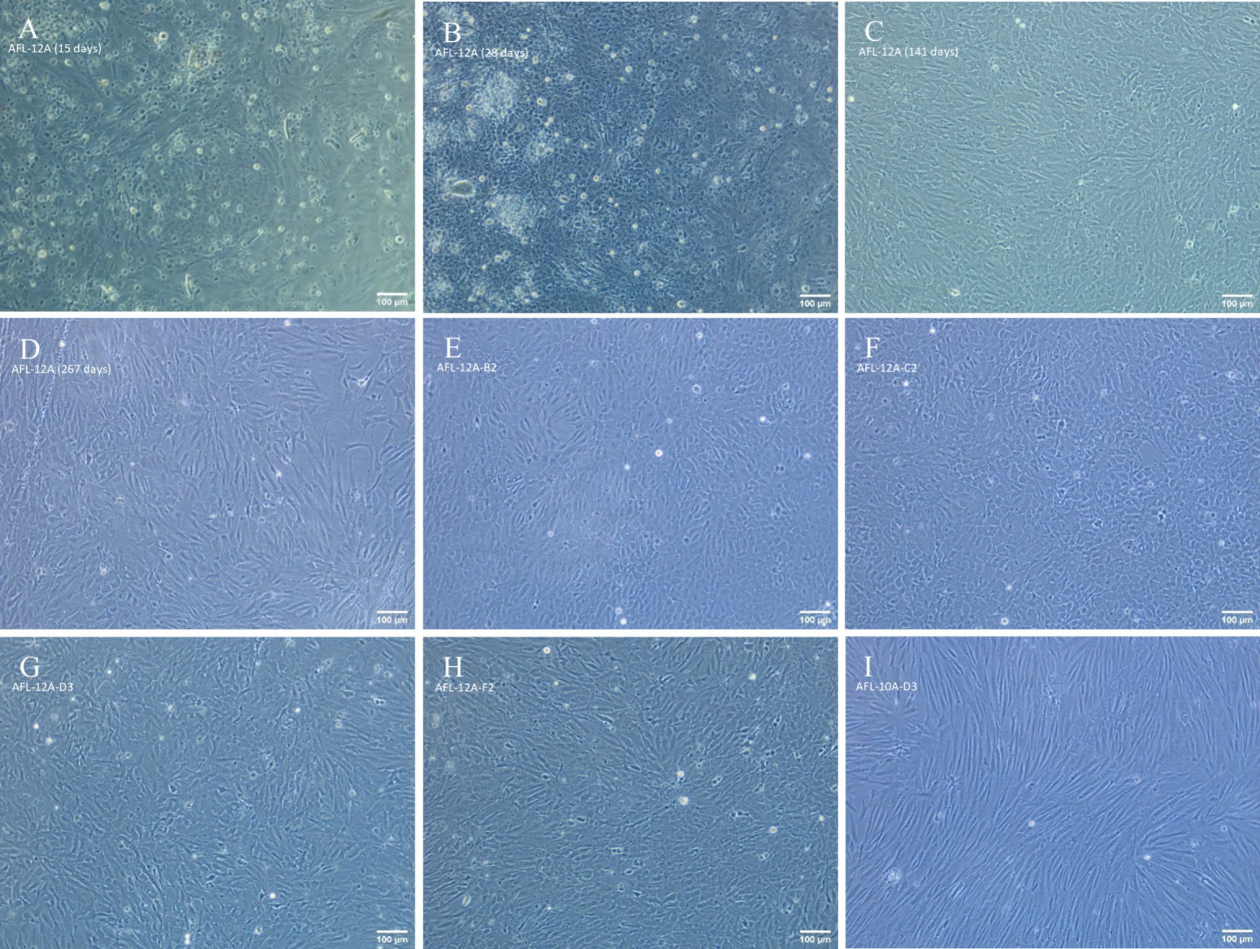
Progressive images of morphology of AFL line 12A monolayers derived from mixed primary-cell populations at increasing passage numbers (*A*-*D*), and resultant monoclonal monolayers of clonally expanded AFL 12A and 10A populations derived from a single progenitor cell (*E*-*I*). (*A)* Monolayer of *A.* *fimbria* primary cells at 15 d in culture. (*B)* Monolayer of *A. fimbria* primary cells at 28 d in culture, immediately prior to the first passage. (*C)* AFL 12A cells at 141 d in culture, passage 8 displaying mixed morphology. (*D)* AFL 12A cells at 267 d in culture, passage 22 displaying mixed morphology. (*E)* Monolayer of clonally expanded AFL 12A-B2 cells derived from a single progenitor cell, at day 339 in culture, passage 25 displaying a uniform epithelial-like morphology. (*F)* Monolayer of clonally expanded AFL 12A-C2 cells derived from a single progenitor cell, at day 339 in culture, passage 25 displaying a uniform epithelial-like morphology. (*G)* Monolayer of clonally expanded AFL 12A-D3 cells derived from a single progenitor cell, at day 356 in culture, passage 24 displaying a uniform epithelial-like morphology. (*H)* Monolayer of clonally expanded AFL 12A-F2 cells derived from a single progenitor cell, at day 356 in culture, passage 24 displaying a uniform epithelial-like morphology. (*I*) Monolayer of clonally expanded AFL 10A-D3 cells derived from a single progenitor cell at day 350 in culture, passage 10 displaying a uniform fibroblast-like morphology. Phase contrast microscopy of AFL cells at × 100 magnification.

Seven monoclonal sablefish cell lines, with either an epithelial- or fibroblast-like morphology, were successfully generated through limiting dilution of polyclonal lines (Table 3). Four of these monoclonal lines were established from polyclonal line AFL-12A, a fifth monoclonal line progenerated from AFL-10A, and two monoclonal lines progenerated from AFL-5A. While the progenitor polyclonal lines exhibited mixed epithelial and fibroblast-like cell morphologies, 10A had a preponderance towards fibroblast-like cells and 12A and 5A had a higher proportion of epithelial-like cells. Indeed, the monoclonal line arising from 10A, namely AFL-10A-D3, exhibited a uniform elongated fibroblast-like morphology while the monoclonal populations from AFL-12A and AFL-5A were more akin to rounded epithelial-like morphologies (Fig. 4*E*–*I*). The four monoclonal lines derived from AFL-12A (AFL-12A-B2, AFL-12A-C2, AFL-12A-D3, and AFL-12A-F2) each retained a uniform epithelial-like morphology after 13, 14, 10, and 11 passages respectively. The two monoclonal lines derived from AFL-5A (AFL-5A-A1 and AFL-5A-B2) retained a uniform epithelial-like morphology after eight passages each. The single monoclonal line derived from AFL-10A, AFL-10A-D3, retained a uniform fibroblast-like morphology after 11 passages. Each polyclonal progenitor retains a mixed-morphology population with one predominant morphology.

**Table 3.** Summary of polyclonal and monoclonal cell lines established from *Anoplopoma fimbria* yolk-sac larva (*AFL*)

Progenitor cell tissue source	Established polyclonal line	Established monoclonal line	
		Epithelial-like	Fibroblast-like
*Anoplopoma fimbria* yolk-sac larva	AFL-5A	AFL-5A-A1 AFL-5A-B2	-
	AFL-6A	-	-
	AFL-9A	-	-
	AFL-10A	-	AFL-10A-D3
	AFL-11A	-	-
	AFL-12A	AFL-12A-B2 AFL-12A-C2 AFL-12A-D3 AFL-12A-F2	-

### Polyclonal AFL cell line growth optimization

Optimal temperature assay

**Figure 5.**
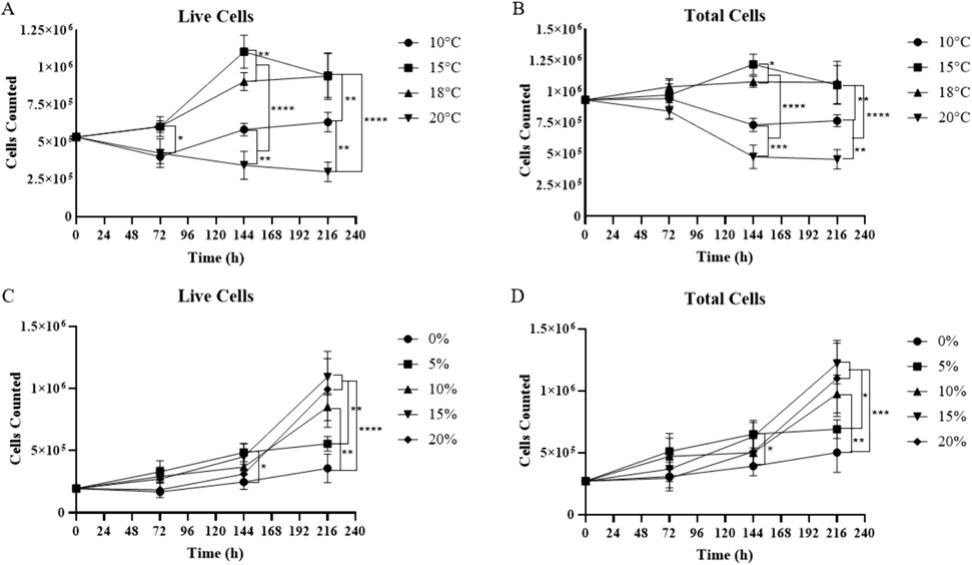
Growth curves of AFL-6A under varying temperatures (*A*, *B*) and serum (*C*, *D*) concentrations. (*A*, *B*) Mean (*n* = 5) counts of live (*A*) and total (*B*) AFL 6A cells seeded in 12-well plates and incubated at 10°C, 15°C, 18°C, or 20°C for 216 h. (*C*, *D*) Mean (*n* = 5) counts of live (*C*) and total (*D*) AFL 6A cells seeded in 12-well plates incubated at 18°C with AFL media containing FBS concentrations of 0%, 5%, 10%, 15%, or 20% (v/v) for 216 h. *Asterisks* represent statistically significant differences between treatments at each timepoint. *Asterisks* indicate the following: *P < 0.05, **P < 0.01, ***P < 0.001, ****P < 0.0001. Statistical significance was determined by Tukey’s multiple comparison post hoc test following one-way ANOVA. *Error bars* represent 1 standards deviation.

Incubation temperature had a significant impact on AFL-6A cell growth (Fig. 5*A*, *B*). At 72 h post-seeding, cells grown at 15°C and 18°C both had significantly greater live counts than at both 10°C and 20°C (ANOVA, live: *F* = 14.05, df = 3, 16, *P* < 0.05) but no significant difference in total cell count. At 144 h post-seeding, cell counts from each temperature differed significantly from all other temperatures, notably cells grown at 15°C had significantly greater live and total counts as compared to 18°C (ANOVA, live: *F* = 86.11, df = 3, 16, *P* < 0.01; total: *F* = 109, df = 3, 16, *P* < 0.05), while cells grown at 20°C showed continued decline. At 216 h post-seeding, there was no significant difference in the number of cells between those grown at 15°C or 18°C; however, cells grown at 10°C and 20°C both showed significantly lower counts than 15°C or 18°C (ANOVA, live: *F* = 34.45, df = 3, 16, *P* < 0.0001). AFL-6A cells incubated at 10°C showed an initial decline in live and total cells but recovered above initial seeding density after 144 h in culture. AFL-6A cells grown at 20°C showed a decline in both live and total cells with no recovery over the duration of the experiment.

Optimal serum concentrations

The concentration of serum within the media did impact AFL-6A cell growth (Fig. 5*C*, *D*). While at 72 h, there was no difference in live or total cell counts, at 144 h, cells grown in L-15 media supplemented with 5% and 15% FBS showed significantly greater live and total cell counts than 0% FBS, while L-15 + 5% FBS showed significantly greater live cell counts than L-15 + 20% FBS (ANOVA, live: *F* = 6.213, df = 4, 20, *P* < 0.05; total: *F* = 4.927, df = 4, 20, *P* < 0.05). At 216 h, there was no significant difference in live or total cell count between cells grown in 0% or 5% FBS supplemented media, nor was there any significant difference in cell count between cells grown in 10%, 15%, or 20% FBS supplemented media. Live and total cell counts of cells grown in media supplemented with 15% or 20% FBS were significantly greater than cells grown in 0% or 5% FBS supplemented media. Cells grown in L-15 media supplemented with 10% FBS showed significantly greater live and total cell counts than cells grown in 0% FBS supplemented media (ANOVA, live: *F* = 16.49, df = 4, 20, *P* < 0.01; total: *F* = 12.35, df = 4, 20, *P* < 0.01).

### Cryopreservation and revival

Polyclonal cell lines AFL-5A, 10A, and 11 cryopreserved in AFL media + 5% DMSO were viable following revival after 14-d cryopreservation in liquid nitrogen. Cells displayed no alterations in morphology or growth rate upon revival. Furthermore, both morphologies of AFL cells present in polyclonal cultures, epithelial-like and fibroblast-like, remained viable after cryopreservation and display no changes in morphology or growth rate. Cryopreserved polyclonal lines AFL-5A, 10A, and 11 recovered to a full monolayer within 2 wk post-revival. Cryopreservation in 100% and 50% Synth-A-Freeze was also viable, however, at roughly half the revival rate of AFL media + 5% DMSO (data not shown). As such, this medium was not considered for subsequent cryopreservation.

### Chromosome analysis

**Figure 6.**
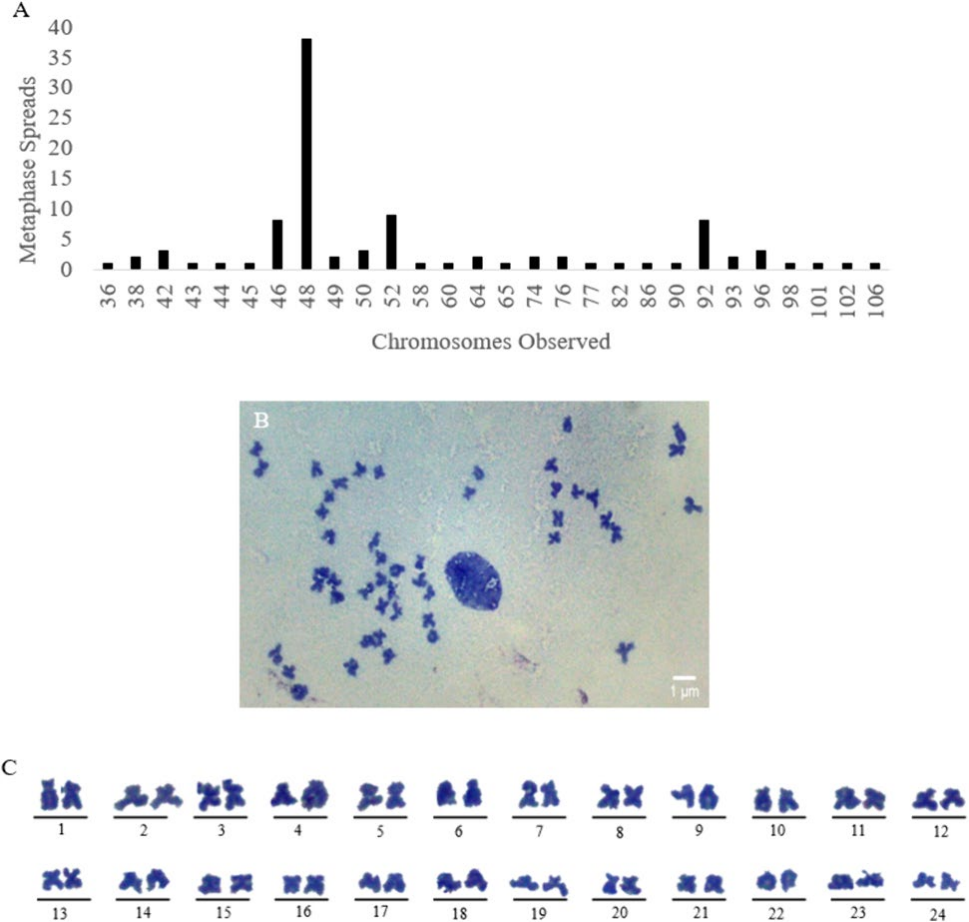
Metaphase chromosome distribution, spread image, and karyotype of AFL-6A cells. (***A)*** Distribution of chromosome numbers observed in 100 metaphase spreads of AFL-6A. (***B)*** A representative light microscopy of AFL-6A cell metaphase chromosome spread stained with Giemsa and observed at × 1000. (***C)*** A representative karyotype of AFL-6A cell chromosomes arrayed by size, stained with Giemsa and observed at × 1000.

AFL-6A cells were diploid with a 2n chromosomal number of 48 (Fig. 6*A*). Counting of 100 metaphase spreads (Fig. 6*B*) revealed a bimodal distribution with the majority of frequencies (55%) clustering around chromosomes 46 to 52 and a smaller cluster (13%) between 92 and 96 (Fig. 6*A*). The modal number (38 of 100 spreads) indicated 48 chromosomes. Arrayed by size, the karyotype of AFL-6A cells shows 24 pairs of chromosomes (Fig. 6*C*).

### Detection of Mycoplasma contamination and molecular confirmation of cell line origin (CO1 Seq)

Each of the six polyclonal AFL cell lines was free of *Mycoplasma* contamination (results not shown) and were confirmed to be of *Anoplopoma fimbria* origin. NCBI BLASTn search queries of a 750-bp sequence fragment of the mitochondrial cytochrome oxidase subunit 1 gene returned matches to only *Anoplopoma fimbria* with identities ranging from 98.42 to 99.68%.

### Sex determination of polyclonal AFL lines

A ~ 300-bp band corresponding to the X chromosome was amplified from all six AFL lines. An additional ~ 900-bp band corresponding to the Y chromosome was amplified from AFL lines 6A, 11A, and 12A. AFL lines 5A, 9A, and 10A display only the X chromosome associated amplicon and therefore an XX female genotype. AFL lines 6A, 11A, and 12A display both the X and Y associated amplicons and therefore an XY male genotype (results not shown).

### Histopathology and larval development

**Figure 7.**
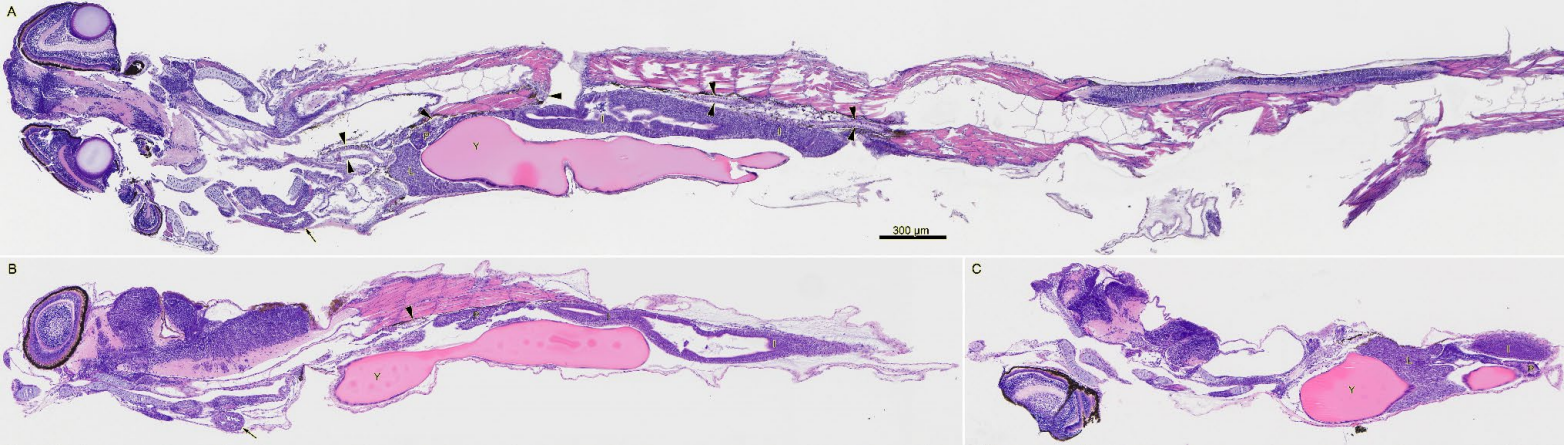
Sagittal sections of *Anoplopoma fimbria* larvae. Heart (*arrows*), kidney (*arrowheads*), liver (*L*), exocrine pancreas (*P*), yolk (*Y*) in yolk sac, and intestine (*I*). (*A)* *A. fimbria* yolk-sac larva age 48 d post-fertilization (dpf). (*B)* *A. fimbria* yolk-sac larva age 27 dpf. (*C*) Same fish as image (*B*), deeper section. Stained by hematoxylin and eosin; magnification is the same for all images.

Larvae 27 or 48 dpf have no evidence of infectious disease. In both larval stages, major organs can be differentiated: heart, liver, exocrine pancreas, kidney, intestine, and yolk-in yolk-sac (Fig. 7*A*, *B*). Other differentiated organs in both larval stages include eye, brain, gill arch, skin, skeletal muscle, spinal cord, and notochord (Fig. 7*A*, *B*). Spleen and gonad were not definitively observed in either larval stage; however, their presence cannot be ruled out in the single section examined through each larva. Larvae in section aged 48 dpf were between 7.1 and 8.8 mm in total length.

## Discussion and conclusions

Here, we report establishing stable cell lines from *Anoplopoma fimbria* derived from a yolk-sac larvae. Since first isolation, six polyclonal cell lines have persisted in culture conditions for more than 18 months and have exceeded 40 passages with no evidence of senescence or reduction in replication. We conclude that AFL cell lines are stable and continuous.

Following the establishment of AFL cell lines, AFL monolayers comprised two main cellular morphologies, a round epithelial-like cell type and an elongated fibroblast-like cell type. To investigate whether this was a morphological difference or perhaps two distinct cell types, both cell morphologies were separated out by serial dilution and clonally expanded to yield uniform monolayers originating from either morphology. Both the rounded and elongated cell types retained their shape and character through clonal expansion and remained as either rounded or elongated. Polyclonal cultures of AFL cells retain both morphologies; however, the rounded epithelial-like cell type predominates over the elongated fibroblast-like morphology. Whether these are different distinct cell types or still simply morphological variations remains unclear. The consistency of morphology throughout clonal expansion and subsequent passaging suggests that the two observed morphologies may be distinct cell types. Further research either through the development of molecular tools for identifying *A. fimbria* cell type and/or using tools developed for other fish species will need to be applied to definitively identify cell type(s). Additionally, whether these mixed cell morphologies are truly stable in the polyclonal lines remains to be determined. Mixed cell morphologies have been observed in other fish cell lines, where in some instances one dominant shape emerged after successive passaging (Fijan *et al*. 1983), while in other cases, mixed cell shapes remained as a stable property of the cell line (Bols *et al*. 2023).

Growth optimization performed on one (AFL-6A) of the six AFL polyclonal lines displayed tolerance for incubation in a range of temperatures with ideal incubation at 15 to 18 °C, while still maintaining viable populations at temperatures as low as 10 °C. Furthermore, AFL-6A showed tolerance for varying concentrations of serum in culture media with no apparent difference in growth between media supplemented with greater than 5% FBS. These findings suggest that AFL-6A possess a robustness for varying incubation and culture conditions which should allow for their use in a wide array of laboratory assays and experiments. While it is likely that the other five AFL polyclonal lines will share similar growth tolerances, differences in cell morphology composition and sex assignments have the potential to vary optimal culture conditions across the six AFL lines. In terms of the revival of AFL cell lines from cryopreservation, AFL-5A, 10A, and 11A stored in liquid-phase liquid nitrogen for 2 wk performed best when AFL media were supplemented with a final concentration of 5% DMSO and frozen at − 1 °C min^−1^. The active cryoprotectant in commercially available Synth-A-Freeze cryopreservation media is 10% DMSO; however, our experience with these three AFL cell lines is that cryopreservation in AFL media + 10% DMSO yields poor revival post-thaw compared to AFL media + 5% DMSO. We suspect this would likely be true across the other three polyclonal lines, although this will need to be specifically evaluated.

Due to the rapid replication of AFL cells, we examined the genetic stability of AFL cells by traditional methods. AFL cells undergo rapid population growth and monolayers are usually confluent approximately 1 wk after passaging at 1:10. *Anoplopoma fimbria* have a well characterized karyotype (Philips *et al*. 2013) with a diploid chromosome number of 48. The metaphase chromosome spreads of each polyclonal AFL cell line revealed the correct number of diploid chromosomes at 48, while experiencing a tetraploidy rate of approximately 11% resulting in 92 or 96 chromosomes. Furthermore, the karyotype of AFL cells shows 24 pairs of chromosomes with no single, triplicate, or more copies of each chromosome. This suggests that AFL cells undergo few chromosome-level errors during replication and are likely genetically stable.

As larvae used in the generation of the AFL cell lines were 45 dpf at time of sampling, their developmental state is best represented by the 48 dpf larva. The presence of eye, brain, skin, skeletal muscle, spinal cord, and notochord at 48 dpf was expected as the eye forms early in development (Kunz 2004), and larvae were motile at time of sampling. The presence of yolk in the yolk sac, as well as intestine, exocrine pancreas, and liver, suggests that at both 27 dpf and 48 dpf, larvae had begun but not yet completed development of the digestive system. The presence of kidney and heart suggests that yolk-sac bearing *A. fimbria* larvae are capable of erythropoiesis and may be capable of leukocyte hematopoiesis as early as 27 dpf (Kunz 2004). Despite the absence of definitive spleen by 48 dpf, larvae may still be capable of mounting an immune response given the presence of other major organs. While the resultant AFL cell lines from those larvae may possess innate immune function if derived from an appropriate tissue, the precise cell type and tissue of origin of AFL cell lines remain unresolved.

All AFL cell lines contained small black granules in suspension in culture media. We suspect that the granules in suspension are released melanin or pigment granules as has been observed in goldfish cell lines (Matsumoto *et al*. 1980). In sablefish, the development and deposition of melanin or pigment begin early in the larval stage (notochord length of 6 mm) preceding the complete absorption of the yolk (Kendall and Matarese 1987; Jensen and Damon 2002). This information suggests that AFL cells may have the capacity to synthesize and release pigment granules as they are yolk-sac larval derived. This may suggest that one or more of the cell types in AFL lines that produce granules are melanocytes, melanophores, or melanocyte-like cells.

In summation, six sublines were established from pools of early yolk-sac larvae (45 dpf), and further characterization of AFL-6A indicates the cell line is tolerant to a range of incubation temperatures and medium serum concentrations, has stable cytogenetics, and can be revived from cryopreserved stocks. The establishment of monoclonal AFL lines of both rounded and elongate cell morphologies, as well as AFL lines composed of male and female genotypes, provides a further breadth of utility. These established and authenticated cell lines will facilitate diagnostics and in vitro research on this important aquaculture species.
